# Ethics and Governance of Neurotechnology in Africa: Lessons From AI

**DOI:** 10.2196/56665

**Published:** 2024-08-29

**Authors:** Damian Eke

**Affiliations:** 1 School of Computer Science University of Nottingham Nottingham United Kingdom

**Keywords:** neurotechnology, Africa, AI, ethics, governance, ethics dumping, regulations, artificial intelligence

## Abstract

As a novel technology frontier, neurotechnology is revolutionizing our perceptions of the brain and nervous system. With growing private and public investments, a thriving ecosystem of direct-to-consumer neurotechnologies has also emerged. These technologies are increasingly being introduced in many parts of the world, including Africa. However, as the use of this technology expands, neuroethics and ethics of emerging technology scholars are bringing attention to the critical concerns it raises. These concerns are largely not new but are uniquely amplified by the novelty of technology. They include ethical and legal issues such as privacy, human rights, human identity, bias, autonomy, and safety, which are part of the artificial intelligence ethics discourse. Most importantly, there is an obvious lack of regulatory oversight and a dearth of literature on the consideration of contextual ethical principles in the design and application of neurotechnology in Africa. This paper highlights lessons African stakeholders need to learn from the ethics and governance of artificial intelligence to ensure the design of ethically responsible and socially acceptable neurotechnology in and for Africa.

## Introduction

The increasing convergence of neuroscience, engineering, materials science, and emerging technologies, such as artificial intelligence (AI), robotics, extended reality, and so on, has given rise to a novel technology frontier called neurotechnology. Once dismissed as the stuff of fiction, cutting-edge invasive and noninvasive neurotechnology is now becoming the reality of our time. Significantly intertwined with advancements in AI, machine learning, and deep learning, neurotechnology holds tremendous promise for research and practice. From brain imaging devices that have transformed our understanding of brain structures and functions to neuromodulation and neurostimulation devices that improve the quality of life of people with brain disorders, neurotechnology is revolutionizing our perceptions of the brain and nervous system. It is also becoming a booming industry with growing private and public investments in direct-to-consumer neurotechnologies [1]. This is owing to a number of factors, including the increasing prevalence of brain diseases and the increasing integration of innovation into biomedical research and practice.

In the last decade, many countries and regions have recognized the need for a maintained public investment in brain research as a priority. Some of the publicly funded large-scale brain research projects include the US Brain Initiative (US $3 billion), the EU Human Brain Project (€607 million), China Brain Project (US $746 million), Japan BRAIN/MINDS (US $365,163,41), Australian Brain Alliance (US $500 million), Canadian Brain Research Strategy (US $250.3 million), and Korea Brain Initiative (US $1.2 billion) [2]. These projects and the emerging landscape of public and private funding opportunities have created a global ecosystem where countries in Africa and other developing countries in the Global South are being left behind in brain research and innovation. Public funding for neuroscience research and innovation is almost nonexistent in Africa [3]. Neurotechnology research and innovation are practically being developed in select countries in North America, Europe, and Asia. These are tech devices that are currently being introduced in African contexts and to African consumers. However, as it has been identified in other emerging technologies, such as AI, technology is developed with a specific context in mind and reflects the dynamics of that context. The use of the technology beyond the context it was created for raises concerns, including bias and discrimination. This means that the use of neurotechnology devices in Africa holds potential ethical and legal risks to both individuals and society.

As this technology expands, neuroethics and ethics of emerging technology scholars are bringing attention to the critical concerns it raises [4-6]. These concerns are generally not new, but uniquely amplified by the novelty of technology. They include issues around privacy, rights, human identity, autonomy, and safety. Many of these are already part of the ethics of emerging technology discourse, particularly AI ethics. The unique risks this technology raises have led to calls for adequate regulatory oversight. Global discussions in this regard have gained momentum in the last decade with a focus on ensuring responsible and equitable design and deployment of this disruptive technology. Intergovernmental bodies, such as the Organization for Economic Cooperation and Development (OECD), UNESCO (United Nations Educational, Scientific and Cultural Organization), and the Council of Europe, are playing major roles in this regard. Chile has become the first country to implement legal measures that address the risks of neurotechnologies [7]. The Chilean constitution was recently amended to legally protect citizen’s mental privacy and free will.

However, as more and more countries discuss the ethics and governance of neurotechnology, there is an obvious lack of regulatory oversight and a dearth of literature on the consideration of contextual ethical principles in the design and application of neurotechnology in Africa. This scenario parallels the historical developments within the field of AI and Africa. In order to circumvent the replication of past errors, this study aims to delineate and address these pitfalls through an in-depth examination and analysis of lessons to be learned. This paper highlights prospective insights that the domain of neurotechnology ethics and governance in Africa may derive from the extant body of literature on responsible AI. Stakeholders, such as neurotechnology developers, policy makers, and academic researchers, stand to gain valuable perspectives from these insights. The resultant impact is anticipated to contribute significantly to the responsible design and implementation of neurotechnology devices in Africa. This encompasses the establishment of robust policies and regulations, as well as the provision of guidance for academic discourse within this domain. This paper starts by providing a conceptual clarification of neurotechnology. Then it discusses why Africa should care about neurotechnology and what responsible neurotechnology will mean for Africa. Lessons are then drawn from the available literature. It is important to note that African societies are not considered as monolithic. However, Africa is taken as a continent with common sociocultural values and challenges relevant to the ethics and governance of neurotechnology.

## What is Neurotechnology?

The human brain remains the most complex organ in the human body largely due to its intricate structure and its central role in coordinating all functions and activities of the body. There are many factors that contribute to this exceptional complexity, including its adaptability and plasticity, cognitive abilities, neural network, sensory processing capabilities, motor control, homeostasis, consciousness, genetic complexity, metabolic demand, multilayered structure, and infinite variability. According to Jorgenson et al [8], the goal of comprehensively understanding all these factors “remains elusive, although not from a lack of collective drive or intellectual curiosity on the part of researchers. Rather, progress frequently has been limited by the technologies available during any given era.” Neurotechnology has emerged to provide a greater understanding of the brain and offer solutions to previously understudied brain disorders.

UNESCO defines neurotechnology as a “field of devices and procedures used to access, monitor, investigate, assess, manipulate, or emulate the structure and function of the neural systems of animals or human beings” [9]. It involves the application of engineering principles to the “understanding, engagement, and repair of the human nervous system” [10]. Neurotechnology refers to a set of technologies, rather than a specific technology, that enables direct connection or interface between technical components with the nervous system [4,11]. This interaction with the brain and nervous system raises a variety of ethical and legal risks, necessitating the discussion of the ethics of neurotechnology.

The importance of ethics in neurotechnology is demonstrated in UNESCO’s call for solid governance of neurotechnology design and deployment at the last international conference on the ethics of neurotechnology on the theme “Towards an Ethical Framework in the Protection and Promotion of Human Rights and Fundamental Freedoms” [12]. From noninvasive technologies used to study the brain to wearable or implantable devices, neurotechnology is opening new possibilities to study the nervous system and help diagnose, treat, and prevent brain-related diseases [1,13]. These technologies are currently being developed for diverse uses in clinical and research settings, as well as for everyday life, workplace well-being, and education. In research, the development of neurosensing technologies, such as magnetic resonance imaging (MRI), functional MRI, electroencephalography, magnetoencephalography, positron emission tomography, functional near-infrared spectroscopy, single-photon emission computed tomography, biomarker analysis tools, and invasive intracranial electrodes, has been transformative for studying the brain. These technologies are fundamental for advancing the understanding of the brain because they provide valuable insights into brain function, neurophysiology, and neurological disorders.

Beyond neurosensing, neurotechnology is also being developed for other purposes, including neurostimulation, neuroprostheses, and neurorehabilitation. Neurostimulation technological devices have shown potential in trials to provide therapeutic relief for a number of brain-related disorders, such as epilepsy [14], chronic pain [15], Parkinson disease (Schuepbach et al [16]), obsessive-compulsive disorder [17], depression [18], addiction disorders [19], spinal cord injuries [20], and Tourette syndrome [21]. Neurotechnology encompasses invasive and noninvasive devices that serve as motor [22] or sensory [23] neuroprostheses. These devices work by connecting to or bypassing any damaged neural pathways to restore function or enhance communication in people with stroke [24], spinal cord injuries [25], amyotrophic lateral sclerosis [26], cerebral palsy [27], and traumatic brain injuries [28]. When these technologies are designed to help individuals regain lost functional abilities, improve their quality of life, and promote independence, it is called neurorehabilitation [29]. These include noninvasive brain-machine interfaces for patients with stroke [30] to noninvasive interfaces, such as brain-actuated robotic devices designed to restore the independence of people experiencing severe motor disabilities [31].

Indeed, there is an emerging and thriving neurotechnology industry; an industry that reached a market value of US $11.3 billion in 2021 and is projected to exceed US $24.2 billion in 2027, with an estimated annual growth rate of 14.4% [32]. According to a recent UNESCO report, “the United States emerges as the main place where neurotechnology-related innovations are generated (47% of worldwide IP5 patent applications in neurotechnology), followed by Korea (11%), China (10%), Japan (7%), Germany (7%), and France (5%)” [1]. It is further revealed that out of 1400 identified neurotechnology companies, 50% are based in the United States, 35% in Europe and the United Kingdom, and the rest in Asia [1]. These figures are not surprising, given the level of public funding that brain research and innovation are receiving in the Global North. While investments in neurosensing, neurostimulation, neuroprostheses, and neurorehabilitation are different in size, volume, and level of maturity (neurosensing being the most mature and with the highest investments), there are exponential increases in all aspects of neurotechnology.

## Why Should Africa Care About Neurotechnology?

Globally, the burden of neurological disorders has increased significantly over the past 2 decades, with a significant increase in mortality (36.7%) and disability (7.4%) rates [33]. There is also evidence from literature to show that there is a large geographical variation in the burden of these disorders [33-37]. There is consistent evidence that there is an increasing prevalence of neurological diseases in Africa, putting huge burdens on public health systems [38-40]. Some of these brain-related diseases include stroke, dementia, Parkinson disease, epilepsy, migraine, medication overuse headache, motor neuron disease, cerebral palsy, brain development disorders, peripheral neuropathy, trauma, alcohol-related brain damage, nervous system complications of HIV/AIDS, brain and nervous system cancers, and multiple sclerosis. It also includes psychiatric disorders and mental health diseases, such as depression, anxiety, schizophrenia, psychosis, bipolar, stress, and other behavioral disorders [41,42]. Many of these are preventable (eg, some developmental disorders and strokes) and others are possibly treatable with novel technologies and therapies.

Silberberg and Katabira [43] believe that the increasing prevalence and burden are the results of factors such as “adverse perinatal conditions, malaria, HIV/AIDS and other causes of encephalitis and meningitis, demographic transitions, increased vehicular traffic, and persistent regional conflicts.” In addition to these, there are other factors that increase the impact of neurological disorders in Africa, such as sociocultural and religious beliefs, stigma and discrimination, lack of quality therapies or treatment, and the absence of organized public sector response [40]. Neurological disorders are often neglected or comprehensively ignored in most African societies [44]. Patients in Africa face challenges related to a lack of health care infrastructure and access to specialized services. Many strongly believe that there is no available treatment or therapy for neurological disorders.

Neurotechnology provides hope to African societies struggling with the burden of neurological disorders. It can help bridge the gap in access to neurological care in many underserved communities. They can help with early diagnosis through advanced neuroimaging and diagnostic tools (eg, portable and low-cost electroencephalography machines). Neurorehabilitation tools, such as virtual reality–based therapies [45,46], functional electrical stimulation [47], and telerehabilitation platforms [48], can provide patients access to rehabilitation services in areas with a lack of resources for therapy. A number of neurostimulation devices may possibly provide effective treatment options for patients with epilepsy, while remote computer-based therapies offer possible relief for a number of brain diseases. There are potentially significant opportunities for neurotechnology to have a positive impact on neurological diagnosis, treatment, and rehabilitation in Africa. However, challenges related to cost and infrastructure remain and will need to be overcome.

In addition to clinical support, neurotechnology can also strengthen research in Africa to better understand the epidemiology of neurological disorders, and factors contributing to their prevalence in Africa, as well as improve the global knowledge of the human brain and nervous system. The introduction of neuroimaging data, generated and processed in Africa, can contribute immensely to the global understanding of the brain and its diseases given the genetic diversity in the continent. It implies that brain diseases plaguing the African population will be better understood, raising the likelihood of developing suitable therapies for them. Neurotechnology can also be applied in marketing and consumer research in Africa, especially in the emerging field of neuromarketing. From product testing, pricing, and value perception to emotion analysis and branding, neurotechnology can help companies understand and influence consumer behavior, preferences, and decision-making. Although far-fetched, this can help African businesses to become more competitive in the global market.

Despite the abovementioned potential benefits of neurotechnology, there are also many good reasons for Africa to prioritize other goals and issues, such as cleaning and sanitation, food security, housing and shelter, education, and access to basic health care. This is a valid argument, and governments need to focus more on these basic needs. However, it is important to note that as a technology, neurotechnology is pervasive, becoming increasingly widespread and influential across various aspects of society. While most of these technologies are being developed outside of Africa, they will be used in Africa. In today’s interconnected world, neurotechnologies can spread rapidly across borders through various channels, such as trade, investment, collaboration, and intellectual property agreements. Therefore, the pervasive and ubiquitous nature of this technology makes it hard to neglect.

Furthermore, as UNESCO [12] has observed, the application of neurotechnology triggers a number of critical ethical and legal considerations, including, but not limited to, autonomy, privacy, mental integrity, human dignity, personal identity, and freedom of thought. Given the African sociocultural context and possibilities of using this technology for enhancement, it also raises fundamental questions related to personhood with profound implications for individuals and societies at large. There are also issues around benefit sharing, digital divide, and accessibility and safety concerns. These concerns are amplified by the fact that current neurotechnological systems are being developed with limited data from Africa without consideration of African sociocultural values and principles. For instance, a brain-computer interface that decodes continuous language from noninvasive recordings would have many useful scientific and practical applications [49], but it raises fundamental questions about the privacy of brain data. In a study that in part relies on an AI transformer model, Tang et al [49] claim to have used functional MRI to produce texts of participants’ imagined thoughts. The implications this has on privacy are novel and immensely significant in the face of an evident lack of governance mechanisms to ensure that these technologies are designed in an ethically responsible, socially acceptable, and legally compliant way. But what should responsible neurotechnology for Africa look like?

## Responsible Neurotechnology in Africa

Like other emerging technologies, neurotechnology raises crucial ethical and legal challenges. However, there remains a dearth of policy frameworks and regulations to ensure the development of responsible and trustworthy neurotechnology. During the UNESCO conference on ethics of neurotechnology in Paris on July 13, 2023, participants agreed on the need for a comprehensive governance framework to harness the potential of neurotechnology and address the evident risks it presents to societies. Speaking at the conference, the Assistant Director-General for Social and Human Sciences of UNESCO declared that “…we must act now to ensure it is not misused and does not threaten our societies and democracies” [12]. In the absence of national, regional, and international principles, policies, and governance mechanisms for neurotechnology, the OECD adopted a set of recommendations on responsible innovation in neurotechnology in 2019 [50]. This is the first attempt to set an international standard that aims to guide government agencies as well as innovators to address the ethical, legal, and social challenges that neurotechnology raises. Principles encompassed in the OECD recommendation are promoting responsible innovation, prioritizing safety assessment, promoting inclusivity, fostering scientific collaboration, enabling societal deliberation, enabling capacity of oversight and advisory bodies, safeguarding personal brain data and other information, promoting cultures of stewardship and trust across the public and private sector, and anticipating and monitoring potential unintended use or misuse. While this instrument does not constitute an international treaty, it covers critical challenges and opportunities for better innovation practices through responsibility-by-design approaches. Such a governance approach is needed to protect and promote human rights and fundamental freedoms. It is an approach that requires the integration of relevant values and principles that reflects the contexts within which the technology will be applied.

So far, the discussion on ethics and governance of neurotechnology has neglected narratives, values, principles, and contexts in Africa. African datasets that can inform the design of neurotechnological systems are currently missing from available open-access platforms. Potentially, therefore, neurotechnology devices are being designed without relevant data from Africa, and the field of neuroscience and neurotechnology remains largely dominated by countries in the Global North. The question then is can responsible neurotechnology be achieved in Africa without African values, principles, data, and experts? Neurotechnology cannot be designed and developed in and for Africa without Africans, their data, and without considerations of African sociocultural contexts, needs, expectations, values, and principles. The current debate on ethics and governance of neurotechnology has taken a similar turn that AI ethics took. Therefore, as UNESCO moves to develop a global normative instrument and ethical framework similar to UNESCO’s Recommendation on the Ethics of AI, it is important for policy makers, innovators, and civil society groups to consider these lessons from AI ethics.

## Lessons From AI Ethics and Governance

Neurotechnology and AI share some similarities and differences. It is common knowledge that artificial neural networks draw inspiration from the brain structure and function because they are designed with interconnected nodes that loosely mimic how brain neurons interact [51]. Both AI and neurotechnology also involve some forms of learning and adaptation. Similarly, they both have uses in health care. However, there are differences in implementation, complexity, and function. For instance, AI uses chips and programmed algorithms, and is based on mathematical models, while neurotechnology often interacts directly with biological systems (brains and nervous systems).

Brains use biological neurons with complex chemical interactions, while AI uses silicon chips and programmed algorithms. The implication of these differences and similarities is that both AI and neurotechnology share common aims and challenges, but they also demand distinct approaches, methodologies, and considerations. The convergence of the 2 can potentially lead to breakthroughs in understanding the brain as well as both biological and AI, ultimately providing benefits for society. However, attention must be paid to the risks they raise for which the discourse on ethical considerations of AI is more advanced than in neurotechnology.

In general, neurotechnology can learn valuable lessons from the evolving field of AI ethics and governance to ensure responsible design, development, and deployment. AI ethics debates highlight the need for transparency and explainability in disruptive technological systems to build trust and accountability. Fairness, equity, responsibility, justice, and autonomy are central to AI ethics. These are principles neurotechnology innovators and policy makers need to adopt to ensure that societal needs and contexts are prioritized.

In addition to these, there are also unique lessons for relevant stakeholders designing, developing, and using neurotechnology in and for Africa. These include innovators, neurotechnology industry players, users, and policy makers.

### Epistemic Injustice

Neurotechnology, like AI, is a value-laden technology, but the critical question is, and should be, whose values and social contexts shape the design and development of the technology [52]? For a number of decades, AI design, development, deployment, ethics, and governance were based on Euro-American epistemic foundations. Values and principles often discussed in the context of value-sensitive design largely reflected worldviews from the Global North. Narratives, values, and principles from the Global North were mostly forgotten or ignored [53,54]. Ruttkamp-Bloem [55] argues that Africa’s exclusion in global AI debates constitutes epistemic injustice that cuts across both hermeneutic and testimonial injustice. This includes the exclusion of African academics and AI practitioners and the work they do in Africa. This lack of diversity, especially in the design and development stage, leads to the exclusion of important knowledge and perspectives from underrepresented communities. Epistemic injustice in AI manifests in different forms including increased bias in AI algorithms, exclusion and marginalization, reinforcement of stereotypes, and other unintended harms. In the context of neurotechnology, this can lead to unfair or inaccurate diagnoses or predictions. Responsible neurotechnology, particularly in Africa, ought to be based on the foundation of epistemic justice—a recognition of Africa’s unique contexts, sociocultural and ethical values, principles, and needs. While there might be a need to enhance capacities for neurotechnology design and implementation in Africa, the inclusion of African experts, data, values, contexts, and principles in the design and implementation of neurotechnology is critical to the idea of responsible innovation in neurotechnology.

### Principles Alone Cannot Guarantee Responsible Neurotechnology

As the awareness of the risks of AI has risen, private and public institutions have responded with a “deluge of AI codes of ethics, frameworks, and guidelines” outlining high-level principles and values to guide ethical design, development, and implementation of AI [56,57]. Mittelstadt [56] argues that the increasing ecosystem of AI ethics has mostly produced, “vague, high-level, principles, and value statements which promise to be action-guiding, but in practice provide few specific recommendations and fail to address fundamental normative and political tensions embedded in key concepts.” The argument here is that AI policy statements and ethical principles have remained ineffective [58] and are largely ignored in many technology-based companies [59]. As Baker and Hanna [60] observed, big technology corporations’ “commitments to ethics are hollowed out by vagueness and legal hand-wringing—in practice, they’re often merely commitments to maintaining public image and mitigating future public relations disasters.” While OECD’s principles of responsible innovation in neurotechnology are laudable, these principles are not enough to ensure that innovators and users practically embed relevant values and principles into the design and implementation of neurotechnology. Responsible neurotechnology needs implementable governance mechanisms, that are ethical, legal, and technical. Building on established approaches of translating principles into practice in biomedical sciences, ethics, and governance of neurotechnology should be more robust and rigorous than what we have observed in AI ethics.

### Possibilities of Ethics Dumping

Ethics and governance of these technologies help to anticipate potential risks, promote safe innovation and deployment, and prevent use that violates core values or exposes people to unacceptable risks. As AI governance has gained momentum in the Global North, Ruttkamp-Bloem [55] believes that Africa has become the ethical dumping ground of the main players on the AI technology scene because of weak regulations. Ethics dumping here refers to the practice of carrying out unethical or legally nonpermissible research activities in countries or regions with weak or nonexistent regulations or governance frameworks. In AI, there is emerging evidence of ethics dumping in the form of “health data colonialism,” in which AI researchers and developers from big technology companies collect data from developing countries to build algorithms in these countries to avoid stricter regulations in their countries [61]. Another example is the outsourcing of data labeling by OpenAI to Africa in what has been called labor exploitation and “unethical outsourcing” [62].

These are possible scenarios that can happen with neurotechnology. As countries in the Global North continue to discuss possibilities of neurorights and governance of neurotechnology, there is a likelihood that neurotechnology companies will exploit the nonexistent regulatory framework in Africa, from unethical human testing to labor exploitation. Africa needs to be aware of this and become proactive in considering governance mechanisms to guide the design, development, and deployment of neurotechnology.

### Diversity of Datasets Is of Critical Importance

The diversity of datasets in the design of emerging technologies is of paramount importance. Diverse datasets offer a more accurate representation of the real world and help to ensure that the technology is fair, robust, inclusive, effective, and sustainable. Fairness, equity, and generalizability in AI mostly depend on how representative the data used to train the AI system are. Nonrepresentative datasets infuse bias into the system. It is also common knowledge that without datasets from AI, AI cannot work for Africa. The quality, quantity, and diversity of data from Africa play a crucial role in the development of accurate, reliable, and generalizable AI systems in Africa. This is the same with neurotechnology. Racially exclusionary practices have been attributed to nanotechnological devices used for neuroimaging, both in the acquisition and analyses [63-65]. Many electroencephalography devices are simply not designed with black hair in mind, which creates implicit racial bias. This means that these devices were designed with insufficient data from black people. It is important for neurotechnology innovators to focus on using sufficient and relevant data from Africa in the design. Africa needs to focus more on generating and having ownership of Findability, Accessibility, Interoperability, and Reusability (FAIR) datasets that can contribute to the design and development of neurotechnology in and for our societies [66].

### Inadequate Regulatory Frameworks

One lesson from the recent AI boom in Africa is that many countries still lack a strong regulatory framework to address the challenges that emerging technologies like AI raise. While existing regulatory frameworks, such as data protection regulations, provide potential channels for integrating regulatory aspects of AI, the rapid advancements of this technology have outpaced the scope of these laws. The European Union’s AI Act has shown that to accommodate the dynamics of such a disruptive technology, a dedicated regulatory mechanism is required. Neurotechnology has been described as a disruptive innovation that will disrupt existing practices as well as traditional boundaries between medical therapies and consumer markets [67]. It has the potential to cause profound social and legal disruption. Owing to their increased capabilities aided by improved computational ability, machine learning, AI, and the availability of large-scale open-access databases, these technologies have the potential to become critical to future legal systems. There are possibilities of using them to predict the likelihood to recidivate, assess volition and intent, detect lies, as well as the potential to reduce recidivism [68]. There are concerns related to mental privacy and surveillance (especially workplace mental surveillance), issues related to equity, and other aspects of personal liberties, which may not fully be captured by existing regulations. Unlike in the case of AI, Africa does not need to play catch up. Relevant stakeholders, including policy makers and researchers, should be proactive in scrutinizing advances in neurotechnology. The time to act is now. There is a great need to develop an effective regulatory or governance framework that can promote responsible neurotechnology in a way that safety, ethical, and legal concerns are sufficiently addressed.

Regulations are important here because this is a technology that challenges existing laws and belief systems. There have been claims in the literature that the risks neurotechnology poses to fundamental freedoms of thought and expression demand new regulations to protect cognitive liberties [69] or neurorights [70]. The risks are significantly exacerbated by the increasing application of neurotechnology in the military as well as the consumer market for digital phenotyping, emotional information, neurogaming, and neuromarketing. These use cases highlight the possibilities of exerting control over brain activities and individual thoughts, which raise the risks of dual use of concern, digital mental surveillance, misuse of neurodata, and other privacy issues. As AI has shown [71], this technology surpasses the ability of existing laws, including data protection laws, to govern its design and development. This paper certainly does not make a case for neuroright laws but seeks to highlight the need to establish regulatory frameworks or amend existing ones that can make neurotechnologies align with African societal values and contexts.

### Available Data Protection Regulations Are Inadequate

The global landscape of data protection regulation is significantly expanding, driven by increasing awareness of privacy issues and the need to regulate the growing digital ecosystem. Most importantly, the introduction of the European General Data Protection Regulation in 2018 is greatly influencing the global approach to data protection with its stringent requirements and extraterritorial scope. So far, over 30 African countries have established data protection laws and or regulations. It is important, however, to note that while data protection regulations play a crucial role in safeguarding the privacy of the individuals, the unique challenges posed by neurotechnology require additional measures and considerations [72,73]. Yuste [74] have raised awareness of the ability of implantable and nonimplantable neural devices to record and alter brain data in ways that jeopardize personal neuroprivacy. There is evidence in the literature to suggest that these devices can successfully decode mental imagery, emotions, story interpretation, and speech [49,75-77]. There are apparent voids in existing data protection regulations to address some of the complex issues involved here. They are not fully aligned to address specific risks and implications associated with the collection and application of brain data by neurotechnology.

To address this gap, some have proposed the establishment of novel human rights, neurorights [70,78], due to concerns around mental privacy, mental integrity, and cognitive liberty. Others have proposed a data-centered approach focused on revising data protection regulations to incorporate issues raised by neurotechnology [79]. As the landscape of neurotechnology continues to progress in Africa, it is important for African policy makers to understand that the available regulations and laws on data are not adequately equipped to address the complex ethical and legal issues neurotechnology raises. While the option of novel human rights is globally being discussed, the data protection–centered approach may be the most pragmatic approach to address the immediate, data-related risks involved in neurotechnology, given the claim for the exceptionalism of neurodata [80].

### The Need for Stakeholder Engagement

At present, the debate over AI governance and regulation in Africa is being shaped by scientists, lawmakers, and scholars in the humanities and the social sciences. However, such debate tends to often lack representation from key stakeholders, which are citizens and community members, who are using these technologies and who will be subject to these new rights. There is a growing consensus among scholars, national governments, and technology corporations about the need to recognize and involve the public as active participants in the design of AI governance [81]. This is often discussed as the democratization of AI or algorithms [82]. AI can have profound impacts on culture, society, and citizens’ rights. Public engagement ensures that these impacts are proactively considered and that the established governance mechanisms reflect the values and priorities of those who will use them. Similarly, ethics and governance of neurotechnology in Africa will benefit greatly from public engagement, not only to raise awareness and understanding of the technology but also to inform the development of governance frameworks that are responsive to the needs and concerns of the public. Public engagement broadens the range of voices that can provide insights to better anticipate potential risks of the technology. It is important that such public engagement exercises are established to ensure public trust which is critical to technology acceptance.

### The Possibility of Corporate Capture

In the absence of functional governance frameworks for responsible AI, technology companies have taken the lead by funding most of the global AI ethics research. This provides an opportunity to influence the research agenda [83]. This is what Gerdes [84] called corporate colonization of the AI ethics research agenda. Large parts of the global AI ethics research are funded by big technology companies fundamentally more interested in their profits rather than public interest [85]. Gerdes [84] also identifies conflicts of interest in AI public policy–making initiatives. Leveraging weak or nonexistent funding mechanisms, regulations, and institutions in Africa, there is a possibility for big neurotechnology companies to control research on ethics and governance of neurotechnology in Africa. Neurotechnology industry players can capture the narrative or discussion on the ethics of neurotechnology to their own benefit. This will have grave consequences in real life. The ethics and governance of neurotechnology, particularly in Africa, need multistakeholder engagement and less performative efforts from policy makers and innovators. It also needs independent (free from the big technology influence) research efforts that will not only inform governance but that can build a sustainable human and technical infrastructure in Africa.

### Dangers of Overly Anthropomorphizing Technology

Human-technology interactions have shown that there is always a tendency to anthropomorphize technology [86]. Indeed, anthropomorphism has become part of the AI literature [87-89]. This is the attribution or projection of humanlike characteristics to inanimate objects, animals, and in this case technology [90]. This is a cognitive bias [91] informed by sociocultural awareness and beliefs. Anthropomorphizing AI can lead to unrealistic expectations and overtrust of the technology. It can blur the lines between humans and machines and consequent attribution of moral agency to machines. As a technology that can interface between the brain and computers, certain neurotechnological devices are developed to create more humanlike interactions. This raises the possibility of overly anthropomorphizing the technology, particularly in Africa, where anthropomorphism is already part of the cultural fabric through religion. This can lead to misconceptions of their capabilities and limitations, raising ethical and practical concerns. To mitigate the negative impacts of the anthropomorphism of neurotechnology, innovators and policy makers need to choose between creating user-friendly neuro-interfaces and maintaining transparency about the nature, capabilities, and limitations of the technology. This includes the education of relevant stakeholders on the roles, nature, and constraints of neurotechnology.

## Conclusion

This paper makes an argument that neurotechnology is no longer a future technology; it is here and is now available not only for clinical research and practice but also to consumers. It is revolutionizing our understanding of the brain and its diseases; providing much needed therapies for a wide range of patients are increasingly used in direct-to-consumer products. Some of these technological devices are being designed in and for Africa [92]. However, the rapid advancement of this technology raises serious risks concerning safety, privacy, human rights, digital divide, bias, and discrimination. With evident weak or nonexistent ethical and regulatory institutions capable of ensuring responsible development and use in Africa, individuals and the society at large face serious risks. Without putting Africans and their needs, interests, values, principles, contexts, data, and expectations into consideration in its design and governance, neurotechnology risks discriminating against Africans as well as jeopardizing the privacy and safety of citizens. This is similar to what is happening in the field of AI. Stakeholders, including policy makers, innovators, and users, can learn the above lessons from AI ethics and governance to ensure that proactive actions are instituted to mitigate against the risks neurotechnology presents to Africans. These lessons need to be taken into consideration as public debates and governance mechanisms for neurotechnology are shaped in Africa. Proactivity and collaboration are the key to being responsive to the demands of mitigating the risks this technology poses. Researchers and scientists working in Africa also need to focus on providing evidence-based insights that can inform policy and practice. This includes consistently providing users and citizens in general with the awareness of the benefits and risks of neurotechnology, which is becoming the new and disruptive technology frontier.

## Abbreviations

AI: artificial intelligence
FAIR: Findability, Accessibility, Interoperability, and Reusability
MRI: magnetic resonance imaging
OECD: Organization for Economic Cooperation and Development
UNESCO: United Nations Educational, Scientific and Cultural Organization
